# Cytoplasmic Asporin promotes cell migration by regulating TGF-β/Smad2/3 pathway and indicates a poor prognosis in colorectal cancer

**DOI:** 10.1038/s41419-019-1376-9

**Published:** 2019-02-06

**Authors:** Hengcun Li, Zheng Zhang, Lei Chen, Xiujing Sun, Yu Zhao, Qingdong Guo, Shengtao Zhu, Peng Li, Li Min, Shutian Zhang

**Affiliations:** Department of Gastroenterology, Beijing Friendship Hospital, Capital Medical University, National Clinical Research Center for Digestive Disease, Beijing Digestive Disease Center, Beijing Key Laboratory for Precancerous Lesion of Digestive Disease, 100050 Beijing, P. R. China

## Abstract

Previous studies revealed that Asporin (ASPN) is a potential mediator in the development of various types of cancer as a secreted stroma protein, but the function of ASPN inside the cancer cells remains largely unknown. Here, we demonstrated a higher expression level of ASPN in colorectal cancer (CRC) than matched normal tissues, and 25% (2/8) CRC showed copy number variation (CNV) gain/amplification in *ASPN* gene. Both higher ASPN expression levels and *ASPN* CNV gain/amplification indicated a worse prognosis in CRC patients. ASPN can promote proliferation, migration, and invasion of CRC cells, and inhibit apoptosis by activating Akt/Erk and TGF-β/Smad2/3 signalings. Further investigations revealed that ASPN interacts with Smad2/3, facilitates its translocation into nucleus, and up-regulates the expression of Epithelial-mesenchymal transition (EMT) related genes. Rescue assays confirmed that TGF-β signaling is essential for the effects of ASPN on promoting CRC cell migration and invasion. In conclusion, ASPN promotes the migration and invasion of CRC cells via TGF-β/Smad2/3 pathway and could serve as a potential prognostic biomarker in CRC patients.

## Introduction

Colorectal cancer (CRC) is the third leading cancer diagnosed worldwide, with the death rate ranking fourth^1^. Even though colonoscopy has been widely used in cancer screening and leads to a decreasing trend in CRC mortality, it remains the main cancer burden in developed countries^1^. Additionally, along with the economic-social transformation of many developing countries, CRC incidence of those areas has increased significantly during the past 20 years^2^. Early CRC is hard to identify and patients with metastasis exhibited a very poor 5-year survival rate (11.7%)^3^. Thus, the discovery of new biomarkers and identification of new drug targets of CRC is of vital importance.

Transforming Growth Factor-β (TGF-β) involving a complex network of pathways regulates cell proliferation, migration, and other functions^4^. It is widely known that TGF-β activates the phosphorylation of Smad2/3, and regulates expression of metastasis associated genes^5^. Particularly, mutation associated with TGF-β/Smad2/3 signaling was identified as one of the most crucial abnormalities in CRC progression^6^. Recently, many new participants in TGF-β/Smad2/3 pathway have been discovered^7,8^, providing original insights into the discovery of new drug targets and development of diagnostic and therapeutic techniques in CRC.

Asporin (ASPN), firstly identified in 2001, is a member of small leucine-rich proteoglycan (SLRP) family^9^. Nevertheless, ASPN is distinct from other class 1 SLRP family members because of its unique aspartate residues named the D-repeat^10^. ASPN was initially identified as an extracellular secreted protein in the studies of bone and joint diseases, such as osteoarthritis pathogenesis, invertebral disc disease, and hypochondrogenesis^10–12^. Over the past decade, ASPN has emerged as a potential biomarker for various types of cancer. Overexpression of ASPN has been identified in breast^13–15^, prostate^16,17^, gastric^18,19^, and pancreas^20,21^ cancers.

ASPN was suggested to be an oncoprotein in most cancer types, such as prostate cancer, pancreas cancer, and scirrhous gastric cancers, while tumor suppressive effects of ASPN were also described in triple-negative breast cancer^22^. Eric W. Klee et al. demonstrated that ASPN could serve as a serum biomarker for advanced prostate carcinoma in both mRNA and protein levels^17^. Meanwhile, Annie Rochette et al. reported ASPN as a stroma expressed biomarker for prostate cancer, which was correlated with the disease progression^16^. Turtoi A et al. identified the overexpression of ASPN in pancreatic cancer when compared with normal tissue and inflammatory tissues^23^. One microarray analysis using bioinformatic method reported that ASPN might be a potential biomarker for CRC detection^24^. Another study suggested that ASPN could enhance CRC metastasis via EGFR/src/cortactin pathway by activating EGFR as an extracellular factor^25^. However, the detailed mechanism of ASPN in carcinogenesis was largely unknown.

Most studies of ASPN on cancers focused on its function as an extracellular matrix component, which was secreted by cancer-associated fibroblasts (CAFs) and then activated or suppressed the receptors located on cancer cell membrane^9^. Subcellular localization of ASPN in the cytoplasm, even in the nucleus, was also observed in many other studies^15,25^, but its exact biological function inside cancer cells was totally unknown.

Here we investigated the role of ASPN in CRC development and revealed the association between its high expression levels and poor prognosis. Particularly, we showed a pro-migration effect of cytoplasmic ASPN, by directly targeting Smad2/3 in CRC cells.

## Materials and methods

### Patients and tissue samples

Application of clinical materials in this research was subject to approval by the ethics committee of Beijing Friendship Hospital, Capital Medical University. 88 pairs of CRC tissues and their corresponding normal tissues were collected from CRC patients who underwent curative surgery between 2011 and 2016, fixed in paraffin. The median (quartiles) age of CRC patients was 63 (26–83) years. All the 88 pairs of CRC and normal tissues were used in immunohistochemistry (IHC) assay. Additional eight pairs of unembedded CRC and normal tissues were preserved in liquid-nitrogen and subjected to CNV detection assays.

### Cell culture and transfection reagents

HCT-8 and RKO human colon cancer cells were purchased from ATCC and cultured in Dulbecco’s Modified Eagle’s Medium (DMEM) added with 10% fetal bovine serum (FBS), with temperature at 37 °C and 5% CO_2_. Lipofectamine 3000 was utilized in the transfection procedure and the efficacy was confirmed with western blot. ASPN siRNA was obtained from Suzhou GenePharma Co., Ltd. The sequence of ASPN-1 is GGATTTTAAACGATACAAA, and ASPN-2 is GCCTTCAGTAAATGTTCATTA. The scrambled (NC) siRNA sequence is UUCUCCGAACGUGUCACGU. Plasmids encoding ASPN were purchased from Changsha Youbio Co.,Ltd, with full-length ORF of ASPN cloned into pLVX-IRES-Puro-3xFlag vector. Three truncated constructs (full-length, amino acid 1–102 and 103–380) were obtained from Shanghai Sangon Biotech Co., Ltd, with pEGFP-C1 vector was applied.

### Immunohistochemistry (IHC) and immunocytochemistry (ICC)

Tissue slides for IHC were prepared using samples in pairs, H&E staining was applied for diagnosis of sections. Slides were deparaffinized xylene after 1 h at 65 °C incubator and then rehydrated in alcohol. 3% H_2_O_2_ for 30 min was applied to block the endogenous peroxidase activity. Dilution factor of ASPN polyclonal antibody (SIGMA, HPA024230) was 200, and the primary antibody incubation was performed at 4 °C overnight. After incubated in biotinylated anti-rabbit secondary antibody, peroxidase activity was detected with DAB. The final step was counterstaining with Hematoxylin. Normal and tumor IHC slides were scored respectively according to the staining intensity of cytoplasmic and nucleus, and the staining scores were reviewed by two pathologists.

HCT-8 and RKO cell lines were applied for ICC after knockdown or overexpression of ASPN. Cells were seeded on coverslips coated with DMEM in 6-well plates in a density of 2 × 10^5^ cells per well. After incubation for 24 h, cells were washed using PBS for 3 times, following with fixed in 4% paraformaldehyde for 30 minutes. Incubation in 3% H_2_O_2_ for 30 minutes was conducted to block endogenous peroxidase activity, then cells were blocked with goat serum for 1 h. Slides were incubated in primary antibody at 4 °C overnight, and the dilution factor of ASPN (SIGMA, HPA024230) polyclonal antibody is the same as IHC. After treated with a universal secondary antibody, A DAB kit and following hematoxylin were applied to stain the slides.

Intensity of staining was classified into negative (0), weak positive (1), moderately positive (2) and strong positive (3). Proportion of positive cells was measured as follows: negative (0), 1–33% (1), 34–66% (2), 67–100% (3). By addition of two parameters, slides were subdivided into low expression group (0–2) and high expression group (3–6).

### Cell viability assay

1,000 cells were seeded in each well of the 96-well plate after transfection. MTS reagent was added into the wells at the time point of 0, 24, 48 and 72 h. After incubated at 37 °C for 2 h, the cell viability was detected with Enzyme—labelled meter (Spectramax M3, Molecular Devices).

### Apoptosis detection assay

HCT-8 and RKO cell lines were used in apoptosis detection assays. After siRNA transfection for 72 h, cells were digested and resuspended in D’PBS, AnnexinV-PE/7-AAD staining kit (BD Biosciences, San Jose, CA) was utilized for cell staining. The apoptosis rate was detected by FACS after 15 min of staining incubation according to the manufacturer’s protocol.

### Wound healing assay

Cells were grown in 6-well plates to confluence. Three wounds were made in each well using a sterile pipette tip. Washing experiment wells with PBS to clear out cellular debris, then cultured in pure medium without FBS. At the time point of 0h, 12h, 24h, 36h and 48 h, the migration status was photographed at the same location of wells.

### Migration and invasion assay

Migration assay was performed using transwell chamber without Matrigel (8 mm, Corning Costar, USA). 750 µl DMEM with 10% FBS was in each well of 24-well plate. For HCT-8 cells, 1 × 10^5^ cells were suspended in 500 µl serum-free medium and pipette into the chamber slowly, with 2 × 10^5^ cells for RKO cell line. The incubation time is 24 h for HCT-8 cell line and 48 h for RKO cell line. After washed with PBS, Cells on the upper surface of the chamber were cleared out using a cotton swab. Methanol was utilized to fix the cells migrated to the lower surface of the chamber. The nuclei of cells were stained with DAPI, and then photographed with fluorescence. The invasion assay was conducted almost the same as above except that transwell chambers (8 mm, Corning Costar, USA) with Matrigel were applied.

### Immunoprecipitation and western blot

Protein was extracted from cells using lysis buffer (Hepes 50 µM, NaCl 150 µM, EDTA 1 mM, 1% Triton, 10% glycerol) supplemented with protease inhibitor cocktail, centrifuged at 4 °C, 12000 rpm for 30 min. After adding protein A/G agarose and antibody, the mixtures were incubated on rotator at 4 °C overnight. After washing the beads with 500 µl lysis buffer for three times, the beads were added loading buffer and denatured at 99 °C for 10 min. Proteins were isolated by electrophoresis using 10% polyacrylamide gel, then transferred to nitrocellulose membrane. 5% (w/v) milk (non-fat milk powder in TBST) was used to block nonspecific binding sites at room temperature for 2 h. Proteins on nitrocellulose membrane were incubated in primary antibody overnight, antibodies employed in the study were listed in Supplementary Table 1. After washed with TBST for 6 times, blots were incubated with secondary peroxidase-conjugated antibodies for 1 h, the detection of blots was performed with an enhanced chemiluminescence system (BIO-RAD, USA).

### Immunofluorescence staining and phalloidin staining

Slides with cells were fixed in 4% PFA for 15 min, followed by incubated in 0.1% Triton X-100 for 10 min to rupture the membrane. 1% BSA in PBST was utilized to block the samples for 1 h. The cells were incubated in primary antibody mixture (anti-ASPN 1:50, anti-Smad2/3 1:50) at 4 °C overnight. Next, the cells were incubated in the Fluorescent secondary antibody mixture (Alexa Fluor 488 goat anti-mouse IgG, 1:200, Life Technologies; Alexa Fluor 568 goat anti-mouse IgG, 1:200, Life Technologies) for 2 h at room temperature, after washed for three times with PBS. Washed for three times with PBS and one time with DDW, the cells were stained with DAPI (sc-24941, Santa Cruz Biotechnology) and photographed using confocal microscopy (IX83, FLUOVIEW FV1200, Olympus).

ASPN overexpressed HCT-8 cells were stained with FITC-Phalloidin (40735ES75, YEASON) according to the manufacturer’s protocol. Stained slides were observed under confocal microscope, compared with control cells.

### CNV detection assay and qPCR

Eight pairs of CRC tissues and matched normal colorectal tissue were enrolled in the CNV detection assay. Specific primers were designed for CNV qPCR (F: TGAAGGGGTGACGGTGTT; R: TGAATCGTTATTGAAAGGTG). Primers for detection of EMT associated genes are listed in Supplementary Table 2. ΔΔCT was calculated to analyze the results of qPCR.

### Cytoplasm and nucleus extraction assay

Cytoplasm and nucleus extraction Reagents (Thermo Scientific, 78833) was applied in the experiment. Operational procedures were strictly performed in accordance with the protocol provided by the company. Western Blot assays were carried out to detect the protein, in which Lamin B1 was used as the marker of nucleus protein, while GAPDH acted as the marker of cytoplasm protein.

### Enzyme-linked immunosorbent assay (ELISA)

HCT-8 and RKO cells were seeded separately in 6-well plates coated with DMEM + 10% FBS in a density of 5 × 10^5^ cells per well. After 48 h incubation, 0.5 ml cell culture supernatant was transferred to 1.5 ml sterilized EP tubes respectively, followed by centrifuging for 20 min at 3000 rpm. The supernatant after centrifuge was detected using ASPN ELISA kit (BEIJING BOSSBIO BIO-TECHNOLOGY CO., LTD) according to the manufacturer’s protocol. The concentration of ASPN was detected with Enzyme— labelled meter (Spectramax M3, Molecular Devices).

### Statistical analysis

All data were analyzed and visualized by R 3.3.1 software (https://cran.r-project.org/) and GraphPad Prism 5 software. The results are expressed as the means ± SD unless otherwise clarified. Unpaired, two-sided Student’s *t*-test was used to compare data of two different groups for normally distributed quantitative data. Kaplan–Meier curves and Log-rank tests were used to evaluate the difference in survival between subgroups. Cox model was applied for multivariant analysis, in which “backward LR” stepwise regression was used for variable selection. All factors which could be potentially prognostic were included in the multivariate Cox analysis, including age, sex, T stage, N stage, M stage, tumor location and ASPN level. A backward process was adopted to assess whether a factor was an independent predictor of DFS or OS. Only independent predictors were included in the final Cox model. The difference was considered statistically significant when *P* < 0.05 unless otherwise clarified.

## Results

### ASPN is overexpressed in CRC and indicates a worse clinical outcome

**Fig. 1:**
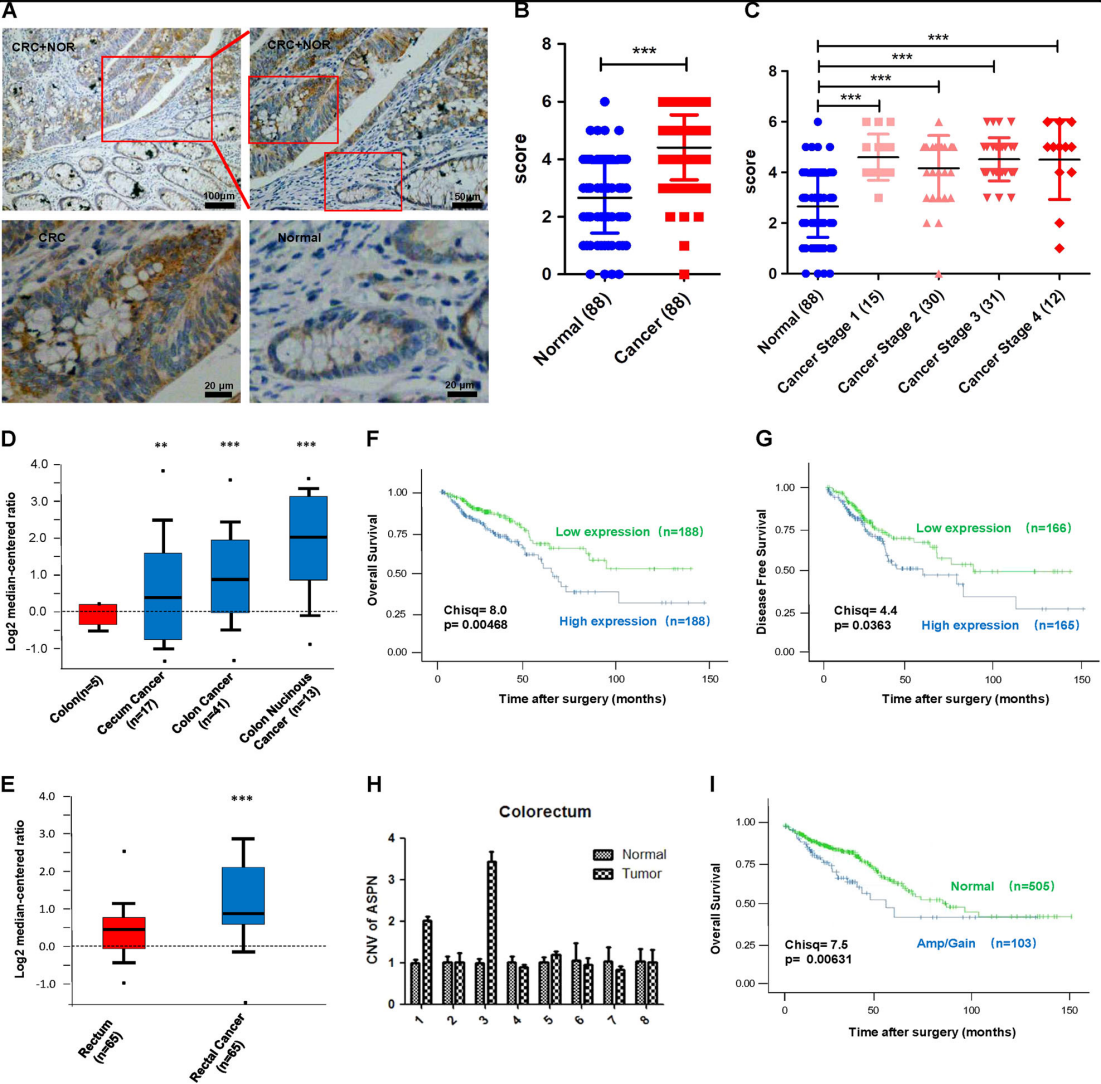
The expression of ASPN in CRC and its association with prognosis. **a** CRC and adjacent normal tissues were immunochemistry stained by 1:200 ASPN antibody. CRC tissues showed strong-positive staining, while normal tissues indicated low ASPN expression. **b** IHC staining score statistics of 88 pairs of CRC and normal tissues. Data were shown as means ± SD. **c** IHC staining scores of four different AJCC stage CRC were analyzed and compared between normal and CRC tissues. Data were shown as means ± SD. **d**, **e** ASPN expression was different between normal colorectal tissues and CRC in mRNA level depending on NCBI Gene Expression Omnibus (D: GSE5261, E: GSE20842). Data were shown in the form of box plot. **f**, **g** The OS (F) and DFS (G) of CRC patients stratified by ASPN expression levels. **h** Twenty five percent (2/8) of CRC tissues were identified with *ASPN* gene CNV gain/amplification. Results are representative of three independent experiments. Values are the mean ± SD of the results. **i** CRC patients with *ASPN* gene CNV gain/amplification exhibited a worse clinical outcome (TCGA CRC dataset). **p < 0.01; ***p < 0.001

To explore the expression pattern of ASPN in CRC, we performed IHC assays in 88 pairs of CRC and their matched normal tissues (the clinical characteristics of the patients were listed in Supplementary Table 3). Meanwhile, we validated the specificity of ASPN antibody by immunocytochemistry (ICC) assays, and the results showed that after the knockdown of ASPN in HCT-8 and RKO cells, the staining intensity of cells were weakened significantly, while the overexpression of ASPN caused a significantly strengthened staining intensity (Supplementary Fig. 1). The results verified that the antibody used in IHC/ICC study is specific to ASPN protein in FFPE samples. IHC staining results suggested ASPN mainly expressed in the cytoplasm and extracellular matrix, and partially in the nucleus (Fig. 1a). Cytoplasmic ASPN of 71.6% (63/88) CRC tissues exhibited significantly higher expression compared with normal controls (*p* < 0.0001, Fig. 1b). Although ASPN level in colorectal normal tissues was significantly lower than CRC of all four different clinical stages, no difference was revealed among the four CRC subgroups (Fig. 1c). Besides, ASPN expression also showed no correlation with age, tumor size, and pathological classification (Supplementary Table 3). To further confirm ASPN is overexpressed in CRC, we extracted data from two independent Gene Expression Omnibus (GEO) datasets^26,27^, which also verified the overexpression of ASPN in CRC at mRNA level (Fig. 1d, e). Analysis of transcriptome data from The Cancer Genome Atlas (TCGA) CRC patients^28^ suggested that higher expression of ASPN was associated with a worse overall survival (376 cases) and disease-free survival (330 cases) (Fig. 1f, g). Univariate and multivariate analysis further confirmed that higher ASPN was an independent indicator for unfavorable prognosis (Table 1).

**Table 1:** Independent prognostic value of ASPN in CRC

		OS		DFS	
		HR(95%CI)	*P* value	HR(95%CI)	*P* value
ASPN	Neg vs. pos	0.444(0.213, 0.926)	0.030	0.426 (0.222, 0.817)	0.010
Age (year)		1.070 (1.037, 1.104)	<0.001	1.025 (0.998, 1.053)	0.065
Size (cm)		1.930 (1.136, 3.281)	0.015	Variable eliminated	
T stage	T3 vs T1-2	Variable eliminated		3.058 (0.929, 10.066)	0.066
	T4 vs T1-2	Variable eliminated		11.886 (3.097, 45.615)	<0.001
N stage	N1 vs N0	2.814 (1.082, 7.322)	0.034	Variable eliminated	
	N2 vs N0	2.931 (1.106, 7.769)	0.031	Variable eliminated	
M stage	M1 vs M0	Variable eliminated		2.810 (1.457, 5.423)	0.002
Overall cox model			<0.001		<0.001

ASPN gene CNV was reported to be associated with other diseases such as acetabular dysplasia^29^. To investigate whether CNV was associated with CRC, we assessed CNV of ASPN by qPCR. The results indicated that 25% (2/8) CRC tissues exhibited an amplification/gain in CNV compared with their matched normal tissues, while the other 6 pairs displayed no significant difference (Fig. 1h). Analysis of CNV data from 608 TCGA CRC patients^28^ suggested that the amplification/gain of ASPN gene CNV indicated a worse clinical outcome (Fig. 1i). Additionally, the prevalence of amplification/gain in ASPN gene in TCGA CRC cohort was 16.9% (103/608), approximately equal to our own data of qPCR assays. Those results suggested that CNV amplification/gain could partially explain the overexpression of ASPN in CRC tissues, and could also serve as a potential prognostic biomarker.

### ASPN promotes cell viability and motility in CRC cells

**Fig. 2:**
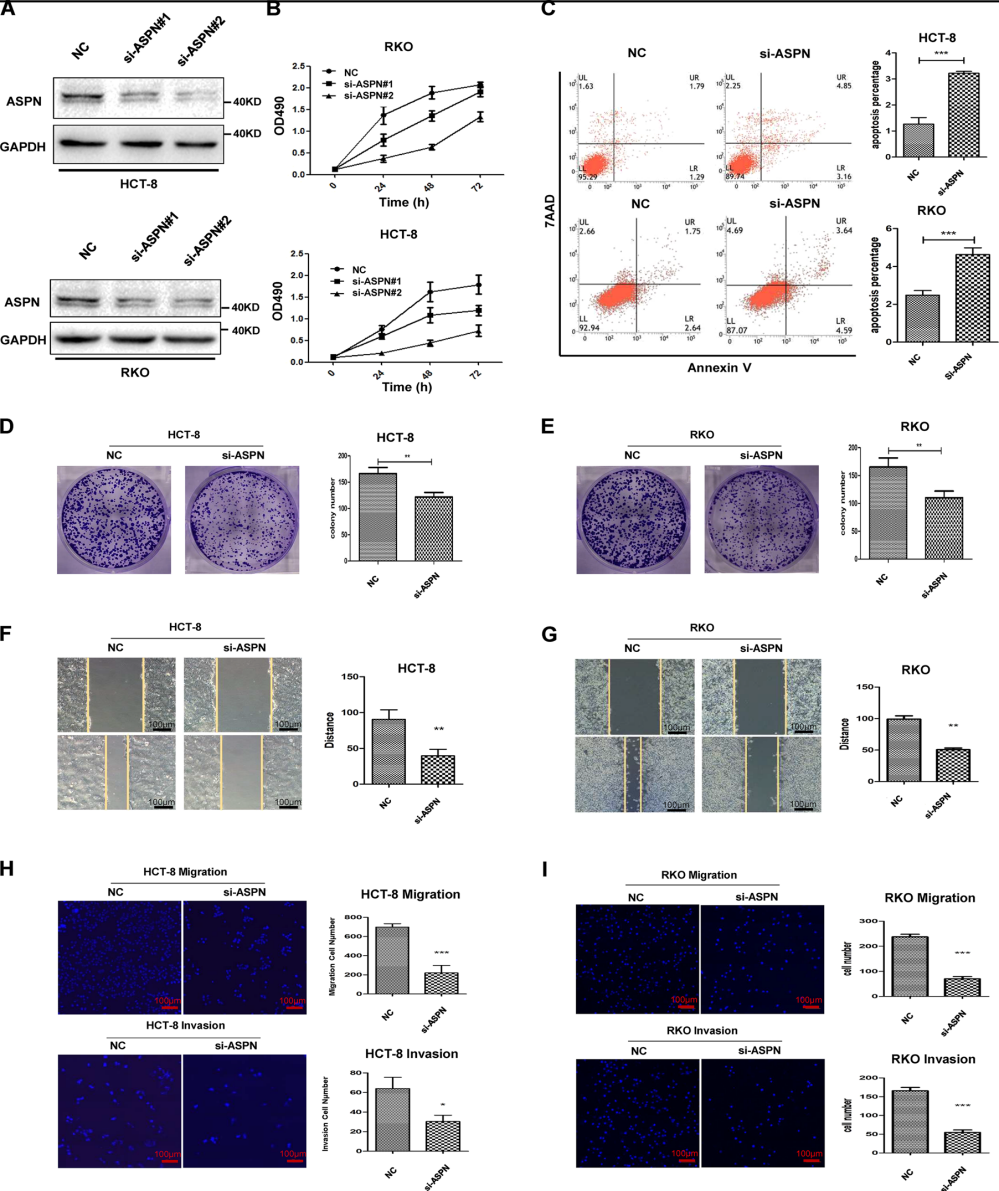
Knockdown of ASPN promotes apoptosis, inhibits cell growth, migration and invasion in CRC cells. **a** ASPN protein expression levels were knocked down by two independent siRNAs in HCT-8 and RKO cell lines. **b** Growth curves of HCT-8 and RKO cells treated with ASPN siRNA or scrambled siRNA (NC). **c** Apoptosis rate detected by FACS after ASPN knockdown in HCT-8 and RKO cells (left panel: representative FACS scatter plots, up, HCT-8; down, RKO; right panel: statistics of early apoptosis cell percentages). **d**, **e** Colony formation assay of HCT-8 (D) and RKO (E) cell lines. **f**, **g** Wound healing assays in HCT-8 (F) and RKO cell lines (G) with ASPN knockdown. Migrated distance was recorded at the time point of 36 h and analyzed in three independent experiments. **h**, **i** Migration (up panel) and invasion (down panel) transwell assays in HCT-8 (H) and RKO cell lines (I) with ASPN knockdown. Cells transferred to the other side of the chamber membrane were stained with DAPI and counted. Results are representative of three independent experiments. Values are the mean ± SD of the results. *p < 0.05;  **p < 0.01; ***p < 0.001

To investigate the role of ASPN in colorectal carcinogenesis, we knocked down the expression of ASPN in HCT-8 and RKO by two siRNAs. The knockdown efficacy was quite extensive, which was examined by Western Blot (Fig. 2a, Supplementary Fig. 2A). MTS assays indicated that the cell viability of ASPN knockdown cells was decreased, both in HCT-8 and in RKO cells (Fig. 2b). As for the colony forming assay, the results showed that ASPN knockdown significantly decreased the colony formation ability of those two CRC cell lines (Fig. 2d, e). To evaluate whether the inhibition on cell growth was the consequence of apoptosis or cell cycle arrest, we assessed the cell apoptosis level and cell cycle by FACS. The results suggested that ASPN knockdown largely improved the level of early-stage apoptosis in CRC cell (Fig. 2c), but had hardly any influence on the cell cycle (Supplementary Fig. 3A, B).

Furthermore, we performed wound healing assays and transwell assays to assess the effect on CRC cell motility of ASPN. Both HCT-8 and RKO with ASPN siRNA knockdown migrated significantly slower than the control (Fig. 2f, g). Transwell assays also suggested that ASPN was positively associated with migration and invasive abilities of CRC cells (Fig. 2h, i).

### ASPN induces mesenchymal morphological changes and promotes progression of CRC via TGF-β/Smad2/3 pathway

**Fig. 3:**
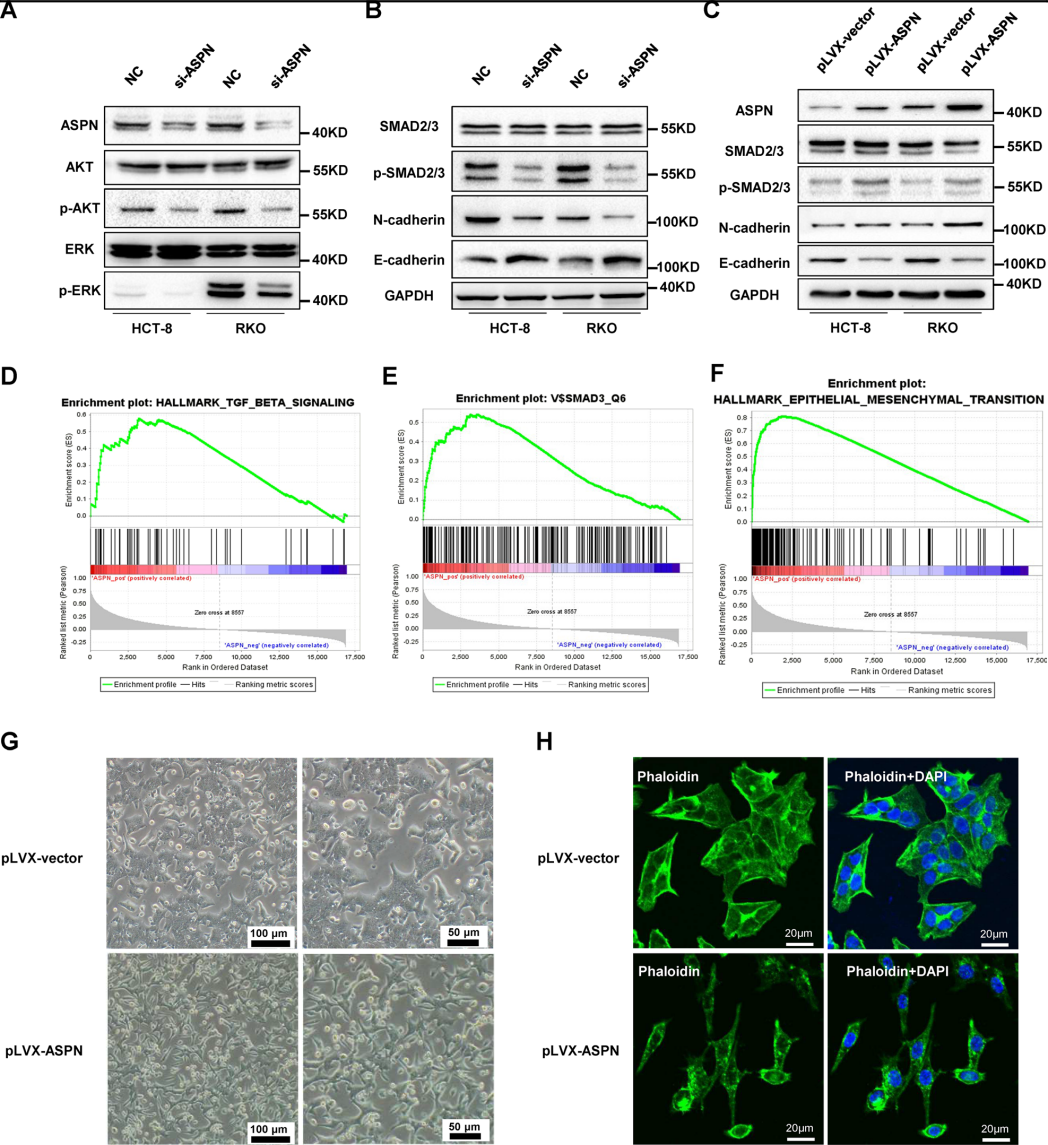
Overexpression of ASPN interferes TGF-β/Smad2/3 signaling and induces morphological changes in CRC cells. **a**, **b** Western blot showed ASPN knockdown down-regulated the phosphorylation of AKT, ERK (A), and inhibited Smad2/3 signaling (B). **c** Overexpression of ASPN elevated the phosphorylation level of Smad2/3, upregulated N-cadherin and inhibited the expression of E-cadherin. **d**–**f** GESA analysis indicated that the expression level of ASPN was correlated with up-regulated genes under TGF-β treatment (D), Smad3 target genes (E) and cell migration associated genes (F). **d**, **e** **f** **g** Morphological change observed in white light after transfected with pLVX-ASPN, compared with control. **h** Morphological characteristics of ASPN overexpression HCT-8 cells observed under fluorescence confocal microscope, compared with control. Results are representative of three independent experiments. Values are the mean ± SD of the results. *p < 0.05; **p < 0.01; ***p < 0.001

It is reported that ASPN could activate EGFR pathway by promoting EGFR phosphorylation in CRC cells^25^. To reveal the underlying oncogenic mechanism of ASPN, we also detected proteins associated with EGFR pathway activation. Our results indicated that in both HCT-8 and RKO cells, siRNA silencing of ASPN resulted in decreased phosphorylated AKT and ERK, whereas the total protein levels of AKT and ERK remained unchanged (Fig. 3a, Supplementary Fig. 2B).

Activation of EGFR alone could not fully result in intense change of cell mobility, thus we also evaluated the change on TGF-β pathway. As showed in Fig. 3b, siRNA silencing of ASPN resulted in a decrease of p-Smad2/3 and N-cadherin, and also an increase of E-cadherin (Fig. 3b, Supplementary Fig. 2C), while ASPN overexpression in CRC cells resulted in an increase of p-Smad2/3 and N-cadherin and down-regulation of E-cadherin (Fig. 3c, Supplementary Fig. 2D). GSEA analysis of transcriptome data from 376 TCGA CRC patients suggested that ASPN level was positively correlated with Epithelial-Mesenchymal Transition (EMT), TGF-β pathway, and genesets targeted by Smad3 motif (Fig. 3d–f), which further confirmed that TGF-β/Smad2/3 signaling pathway was involved in the ASPN-related CRC migration.

TGF-β/Smad2/3 signaling was reported to induce EMT, which was correlated to tumor progression and metastasis^30^. As shown in Fig. 3g, h, overexpression of ASPN was found to induce mesenchymal morphological change in HCT-8 cells, suggested that ASPN would enhance CRC migration by inducing EMT.

### ASPN co-localizes, interacts with Smad2/3 and facilitates its entering into nucleus

**Fig. 4:**
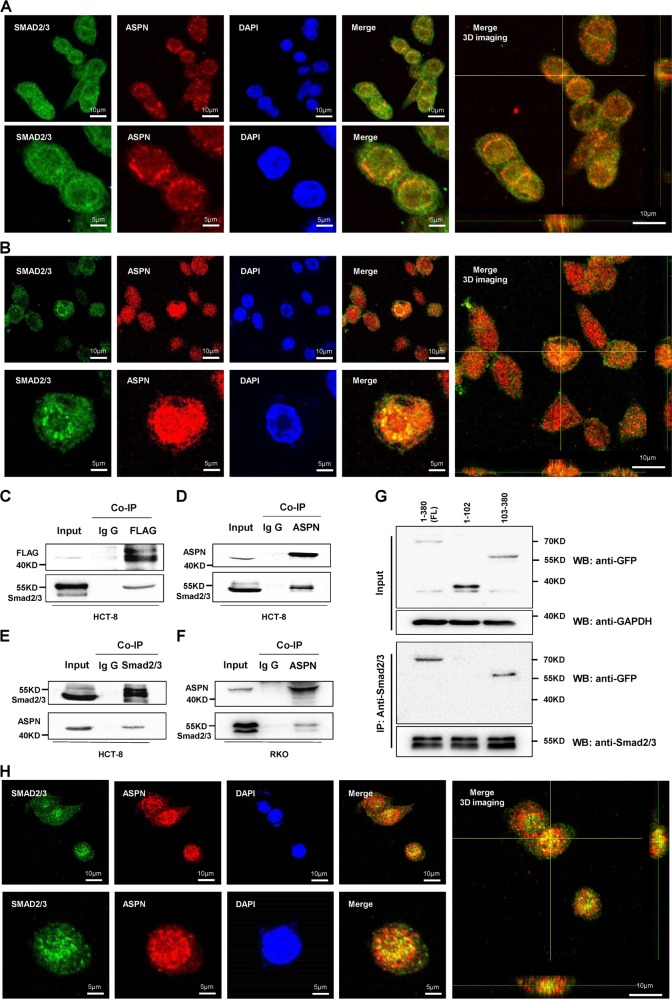
ASPN co-localizes and interacts with Smad2/3 by Leucine-rich repeats, TGF-β facilitates translocation of ASPN/Smad2/3 complex into nucleus. **a**, **b** Cellular immunofluorescence co-localization detected by confocal microscope in HCT-8 (A) and RKO (B) cell lines. ASPN (red) and Smad2/3 (green) perfectly co-localized to form yellow patterns. DAPI was used to stain nuclei, and 3D imaging was also displayed (right panel). **c** Co-IP assays confirmed the interaction between exogenous flag tagged ASPN and Smad2/3 in HCT-8 cells. **d**–**f** Co-IP assays confirmed the endogenous interaction between ASPN and Smad2/3 in HCT-8 cells (D.E) and RKO cells (F). **g** Mapping the domain of ASPN required for its association with Smad2/3. GFP-tagged full-length (1–380) and truncated forms of ASPN were expressed in HCT-8 cells, immunoprecipitation was conducted with an anti-Smad2/3 antibody applied. **h** After treated with TGF-β, more colocalization points of ASPN and Smad2/3 were observed in the nucleus, compared with Fig. 4a, b above

Our previous IHC results indicated that ASPN was not only located in the extracellular matrix as most studies reported^9^, but also localized in cytoplasm, and even nucleus. To confirm this phenomenon and provide more clues to uncover related mechanism, we performed immunofluorescence assays and revealed that ASPN co-localized with p-Smad2/3 in the nucleus in both HCT-8 and RKO (Fig. 4a, b). Furthermore, co-IP assays were also carried out, which further indicated that both exogenous and endogenous ASPN could interact with Smad2/3 (Fig. 4c–f). ASPN consists of 11 leucine-rich repeats (LRRs) domain and 1 LRR N-terminal (LRRNT) domain. Co-IP assays applying truncated forms of ASPN indicated that ASPN interacts with Smad2/3 via LRR domains (Fig. 4g).

In immunofluorescence assays, we noticed that the co-localization signal of ASPN and Smad2/3 was very strong on the cell nuclear membrane, intermediate in the nucleus, but weak in the cytoplasm. Considering only p-Smad2/3 can enter into the nucleus, we further conducted immunofluorescence co-localization assay using TGF-β1 treated HCT-8 cells. The results indicated that TGF-β1 promoted the translocation of ASPN-p-Smad2/3 interaction complex, and more co-localized sites were observed in TGF-β1 treated cells (Fig. 4h), compared with untreated cells (Fig. 4a). Prior studies reported that ASPN was a secreted protein, binding TGF-β1 and blocking downstream signaling^22,31^. Therefore, we also performed ELISA assays and found that ASPN protein could be secreted by both RKO and HCT-8 cells (Supplementary Fig. 4).

**Fig. 5:**
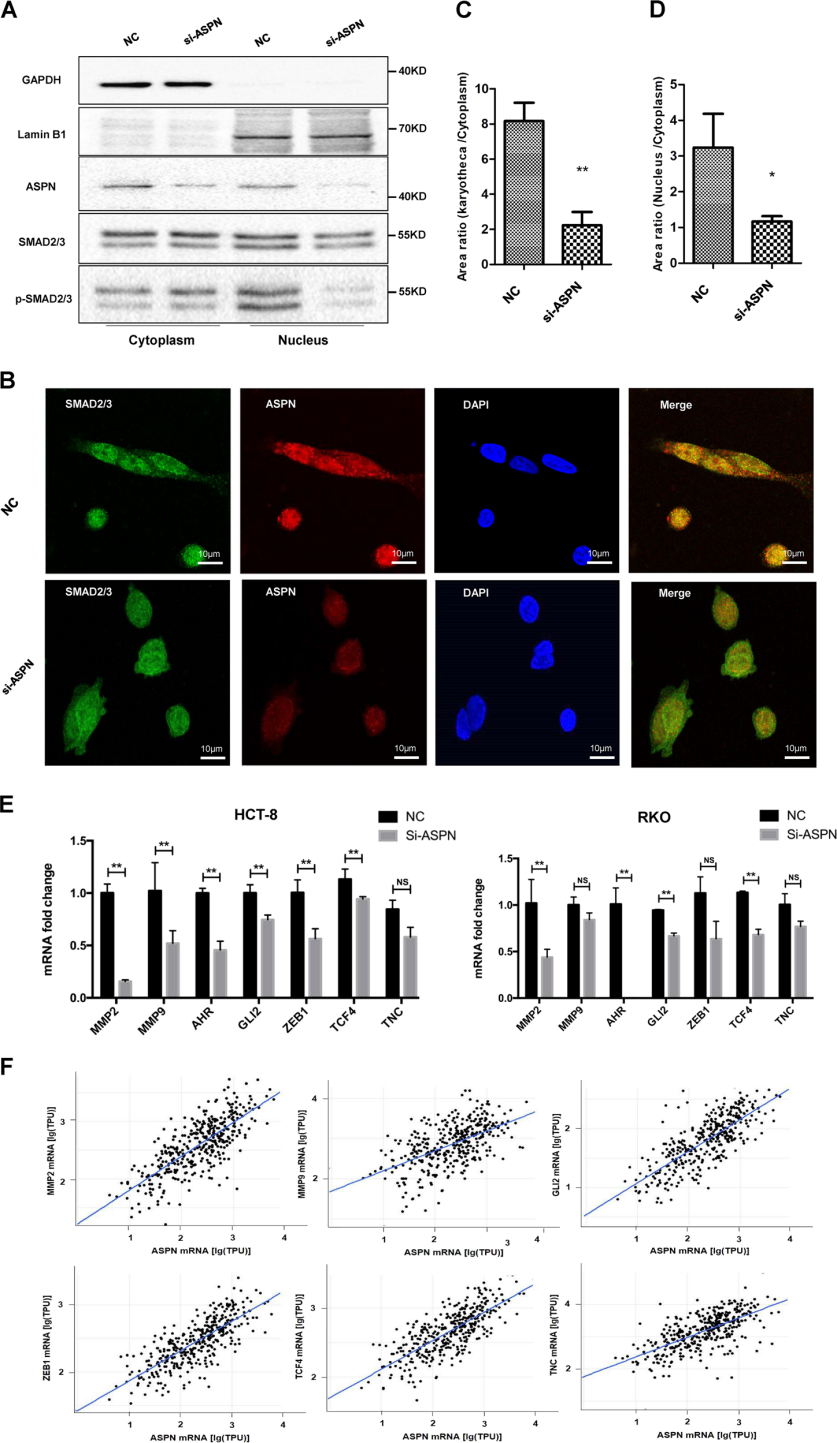
ASPN facilitates Smad2/3 entering into nucleus and participates in the regulation of Smad2/3 targeted EMT-related genes. **a** Nucleus and cytoplasm protein separative extraction assay demonstrates that ASPN facilitate Smad2/3 entering nucleus. GAPDH was used as cytoplasm protein marker and Lamin B1 served for nucleus marker. **b** Immunofluorescence assay indicated both intranuclear and karyotheca Smad2/3 decrease significantly under ASPN siRNA knockdown. DAPI was used to stain nuclei. **c** Statistics of Smad2/3 fluorescence strength ratio (karyotheca:cytoplasm) under ASPN knockdown. **d** Statistics of Smad2/3 fluorescence strength ratio (nucleus: cytoplasm) under ASPN knockdown. **e** ASPN siRNA knockdown decreased the mRNA expression level of EMT-related genes in HCT-8 (left panel) and RKO (right panel) cells. The detected genes by RT-qPCR included MMP2, MMP9, AHR, GLI2, ZEB1, TCF4 and TNC. **f** In silico analysis indicated very strong-positive associations between mRNA expression levels of ASPN and those EMT genes (original data were extracted from TCGA CRC dataset). Results are representative of three independent experiments. Values are the mean ± SD of the results. *p < 0.05; **p < 0.01

Additionally, we evaluated the protein level of p-Smad2/3 in cytoplasm and nucleus separately to clarify ASPN’s effects on p-Smad2/3. The results suggested that the cytoplastic p-Smad2/3 level was unaffected, but the nuclear p-Smad2/3 was decreased (Fig. 5A, Supplementary Fig. 5A). Immunofluorescence assays of ASPN silenced CRC cells and their controls further confirmed ASPN’s effects on subcellular localization of p-Smad2/3 (Fig. 5b). The p-Smad2/3 fluorescence intensity ratio (nuclear membrane/cytoplasm) was largely reduced by ASPN siRNA treatment (Fig. 5c), and meanwhile the fluorescence intensity ratio of nucleus to cytoplasm was also significantly decreased (Fig. 5d). Thus we concluded that ASPN could recruit p-Smad2/3 on the nuclear membrane and facilitates its entering into the nucleus.

### Knockdown of ASPN decreases the expression of EMT-related molecules associated with Smad2/3

Crucial molecules mediating the process of EMT include Snail, Slug, Zinc Finger E-box Binding Homeobox 1 (ZEB1), etc.^30^. In order to explicit whether ASPN promotes CRC invasion via those TGF-β induced EMT proteins, RT-qPCR was applied to evaluate the mRNA fold changes of those proteins under ASPN siRNA treatment. Our results suggested that all those proteins (MMP2, MMP9, AHR, GLI2, ZEB1, TCF4, TNC) were significantly down-regulated when interfering ASPN in both HCT-8 and RKO cell line (Fig. 5e). Additionally, we also extracted and analyzed the data from TCGA and identified very strong-positive correlations between ASPN and those Smad2/3 downstream EMT molecules (Fig. 5f, Supplementary Fig. 6).

### TGF-β signaling is essential for ASPN’s promotion on the invasion of CRC

**Fig. 6:**
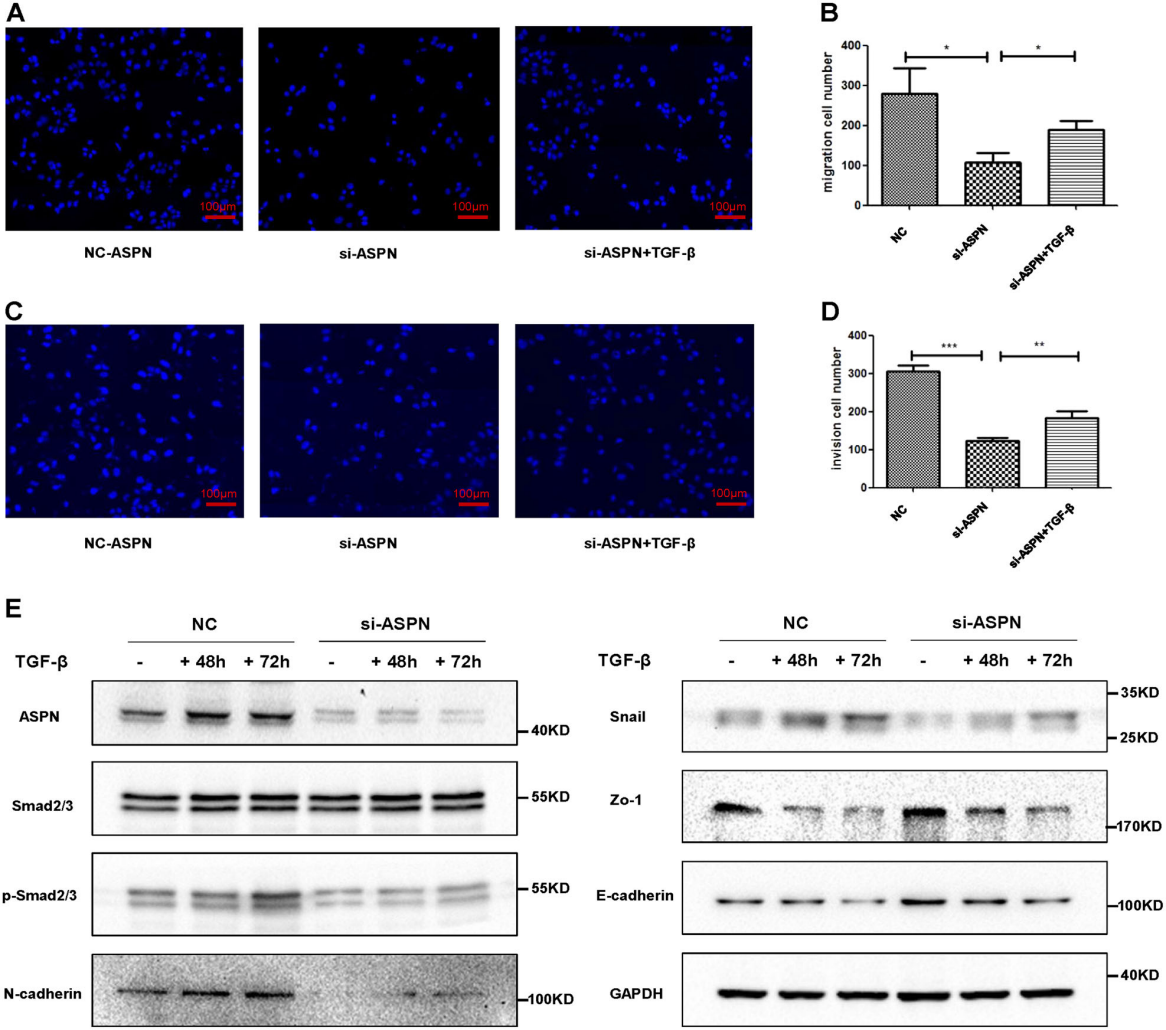
TGF-β signaling is essential for the pro-migration and pro-invasion function of ASPN. **a** After treated with TGF-β for 24 h, the inhibited migration by ASPN knockdown in HCT-8 cells was partially restored. **b** Statistical analysis of three independent migration transwell experiments. **c** After treated with TGF-β for 24 h, the inhibited invasion by ASPN knockdown in HCT-8 cells was partially restored. **d** Statistical analysis of three independent invasion transwell experiments. **e** si-ASPN treated HCT-8 cells displayed a decreased expression level of p-Smad2/3, N-cadherin and Snail, while ZO-1 and E-cadherin increased. After treated with TGF-β, those expression alterations were all nearly fully reversed in a time-dependent manner. Results are representative of three independent experiments. Values are the mean ± SD of the results. *p < 0.05; **p < 0.01; ***p < 0.001

In order to verify the role of TGF-β/Smad2/3 signaling in the invasion ability of CRC promoted by ASPN, rescue assays were performed. By treating with TGF-β, the migration and invasion ability were partially reversed in ASPN knocked-down HCT-8 cells (Fig. 6a–d). Western blot also supported this conclusion with a reversed protein level of p-Smad2/3, N-cadherin, Snail, Zo-1 and E-cadherin (Fig. 6e, Supplementary Fig. 5B, C).

On the contrary, by treating CRC cells with TGF-βR antagonist LY2109761, ASPN-induced migration, and invasion were largely eliminated in HCT-8 cells (Supplementary Fig. 7A–D), emphasizing the role of TGF-β signaling in mediating ASPN-induced invasion of CRC.

## Discussion

The roles of ASPN in the progression of cancer have been reported to be controversial, with cancer promoting or suppressing function displayed even in the same cancer types^13–21^. For example, in breast cancer, ASPN was reported as a fibroblast-derived cancer suppressor through the inhibition of TGF-β1 signaling^22^. However, another study provided the opinion that ASPN held a dual role in the progression of breast cancer^14^. Studies of ASPN in pancreatic, gastric and prostate cancer drew a consistent opinion that ASPN could promote the development of cancer, but the underlying mechanisms were still elusive^16,19,20^.

For colorectal cancer, a few preliminary studies suggested ASPN might be a potential biomarker for CRC detection^24,25^. In our study, a higher expression level of ASPN was observed in the CRC glandular epithelium, compared with matched non-cancerous tissue. The overexpression of ASPN was also verified by two independent cohorts in GEO database in our bioinformatic analysis, which made our conclusion more credible. We also revealed that ASPN level could be a very good predictor of prognosis in CRC patients, which further highlight the potential of ASPN serving as a useful biomarker.

Previously, ASPN gene CNV was only reported in orthopedics diseases^29^. Here, for the first time, we revealed an amplification/gain in CNV in CRC patients, which could partially explain the overexpression of ASPN in CRC tissues. We also suggested amplification/gain in ASPN gene CNV could indicate a worse clinical outcome in CRC patients. Furthermore, when compared with the genes (OGN, ECM2) located nearby ASPN, we found that both aneuploidy gain and amplification of ASPN of CRC patients were due to the change of a large region of chromosome nine encompassing ASPN (not a focal amplification of ASPN).

Additionally, it warrants attention that all previous studies of ASPN were focused on the extracellular function of ASPN, although subcellular localization of ASPN in the cytoplasm, even in the nucleus, was also reported in many other studies^15,25^, the exact biological function of ASPN inside cancer cells was largely neglected. Here we found that besides being located in the stroma of CRC, most of the ASPN protein was located inside colorectal cancer cells, so that the possible intracellular function of ASPN should be given more attention. In this study, combining bioinformatic analysis and molecular assays, we suggested that ASPN interacted with Smad2/3, facilitated its entering into nucleus, and activated the TGF-β/Smad2/3 signaling and EMT, which is the first reported function of ASPN inside cells. However, whether more other signaling pathways were affected by intracellular ASPN still needs further investigation.

In this study, ASPN was shown to promote cell proliferation, migration, and invasion, and reduce the early-stage apoptosis rate, which is partially in consistent with a previous study^18^. Qian et al. suggested that the increase of migration and invasion by ASPN was due to activation of EGFR^18^. Nevertheless, how ASPN activated EGFR was not explained. Here we gave an alternative explanation of ASPN’s pro-motility role in cancer cells, i.e. activation of TGF-β/Smad2/3 signaling from inside cells. Firstly, our bioinformatic analysis indicated a strong association between ASPN and TGF-β/Smad2/3 related pathways. Then, we revealed an interaction between ASPN and Smad2/3, which further increased the proportion of nuclear ASPN in CRC cells. Furthermore, we found that after ASPN knockdown, p-Smad2/3 and its potential EMT-related targets, such as N-cadherin, were largely decreased.

It was reported that phosphorylated Smad2/3 entered the nucleus with the help of Smad4 and co-factors, and acting as transcription factors, including expression of EMT-related genes^32,33^. EMT process has been widely reported to be very critical for the malignant phenotypes’ acquirement of cancer cells, especially the acquisition of high mobility^30^. Wang et al. also reported that ASPN could regulate EMT in pancreatic cancer by interacting with receptor CD44 as an extracellular factor and activating NF-kB/p65 signaling^20^. Thus, we suggested that ASPN could induce EMT and enhance cell migration through two independent pathways. In our study, ASPN was identified as a facilitator in the process of p-Smad2/3 entering into the nucleus, and the enriched localization on karyolemma of ASPN further supported this theory. Thus, we drew a conclusion that ASPN could recruit p-Smad2/3 on the nuclear membrane and further facilitate its transportation into the nucleus.

Additionally, Maris et al. reported that fibroblast-derived ASPN could block the TGF-β1 receptor and inhibit EMT process^22^, which suggested that the extracellular ASPN had a Janus-faced effect in EMT and cell migration by binding to different membrane receptors. In order to determine whether HCT-8 and RKO cell lines secrete ASPN, we conducted ELISA assays for cell culture supernatant of those cell lines. We found that both HCT-8 and RKO cell lines secreted ASPN, which was contrary to the Smad2/3 activating function of cytoplasmic ASPN we identified. However, in this study, we have strictly demonstrated that cytoplasmic ASPN directly interacted with p-Smad2/3 and facilitated the translocation of p-Smad2/3. Overexpression of ASPN promoted EMT and migration of CRC cells, and siRNA knockdown of ASPN exhibited an opposite effect (Figs. 2 and 3). Thus, we suggested that cytoplasmic ASPN had a totally different role in CRC compared with extracellular ASPN, and cytoplasmic ASPN exhibited a dominant role in the development of CRC. Additionally, extracellular ASPN function via directly binding to TGF-β1, thus mutation of TGFBR (which is common in CRC cells such as RKO) could theoretically abort the TGF-β1 inhibition effect of extracellular ASPN. We proved that ASPN promoted RKO cell migration (Fig. 2f–i), suggesting the pro-TGF-β/Smad2/3 role of cytoplasmic ASPN is independent of TGFBR status, which is totally different from the way extracellular ASPN inhibites TGF-β/Smad2/3 pathway. Additionally, we treated HCT-8 (without TGFBR mutation) with TGF-β1 and found the co-localization of ASPN and Smad2/3 concentrated much more in the nucleus (Fig. 4h). The results indicated that TGF-β promoted the translocation of ASPN/Smad2/3 interaction complex into the nucleus, and validated the involvement of TGF-β/Smad2/3 pathway in the ASPN-induced CRC migration. Besides, p-ERK was also found decreased in ASPN siRNA treated cells in our results, so that the crosstalk between ERK pathway and Smad2/3 signaling should be addressed. It was reported that p-ERK phosphorylated Smads in specific sites, and Smads phosphorylated by ERK could accumulate abundantly in the nucleus at a high level of TGF-β stimulation^5^. It is also possible that the increase of ERK phosphorylation level of ASPN contributes to the activation of Smad2/3, and the relationship among ASPN, ERK and Smad2/3 may be much more complicated. Thus, the effects of extracellular ASPN and cytoplasmic ASPN on TGF-β/Smad2/3 pathway were totally different, and possible feedback-regulated, crosstalk and mutual regulatory mechanisms could exist among ASPN, TGF-β/Smad2/3 and other signaling pathways.

In conclusion, our study reveals that ASPN expression is obviously elevated in CRC tissue, which indicates a worse clinical prognosis. As an oncogenic protein in CRC, ASPN interacts with Smad2/3, facilitate its entry to nucleus, induce EMT, and promote cell invasion. Our findings not only provides a new evidence to improve our understanding of the function of ASPN in the development of CRC, but also showed insight into the discovery of a new drug target and the progression of new patient stratification strategies for CRC treatment.

## Supplementary information

Supplementary Figures

Supplementary Tables

## Data Availability

The datasets and materials used for the study are available from the corresponding author on reasonable request.
