# Supplementary material for: Cytoplasmic Asporin promotes cell migration by regulating TGF-β/Smad2/3 pathway and indicates a poor prognosis in colorectal cancer
Source: Cell Death Dis. 2019 Feb 6;10(2):109. doi: 10.1038/s41419-019-1376-9 (PMC6365561; doi:10.1038/s41419-019-1376-9)
Supplement: Supplementary file 1 — Supplementary Figures [file 41419_2019_1376_MOESM1_ESM.pdf]

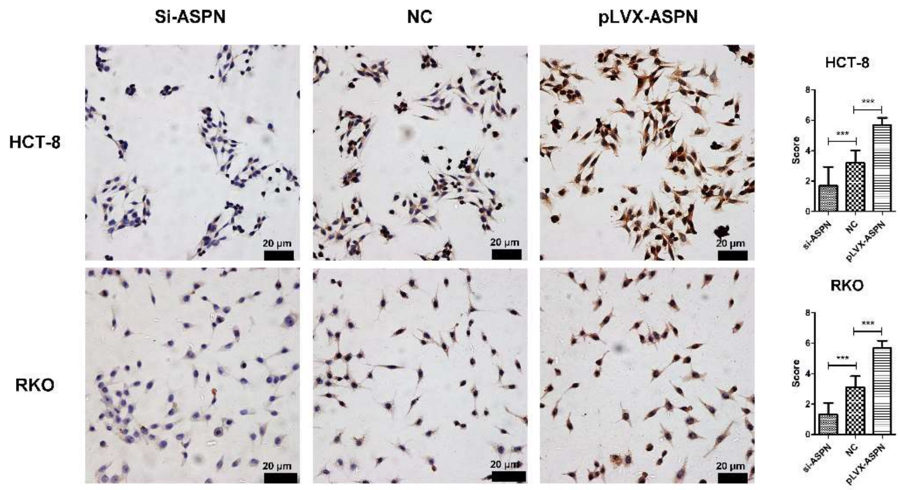

Supplementary Figure 1. ASPN antibody applied for IHC staining is specific to ASPN protein. Immunocytochemistry (ICC) staining were conducted with HCT-8 and RKO cells after ASPN knockdown or overexpression, compared with control (left). Statistics analysis indicates significant difference of staining intensity among the 3 groups (right).Results are representative of three independent experiments. Values are the mean ± SD of the results. * $p < 0.05$; **$p < 0.01$; *** $p < 0.001$.

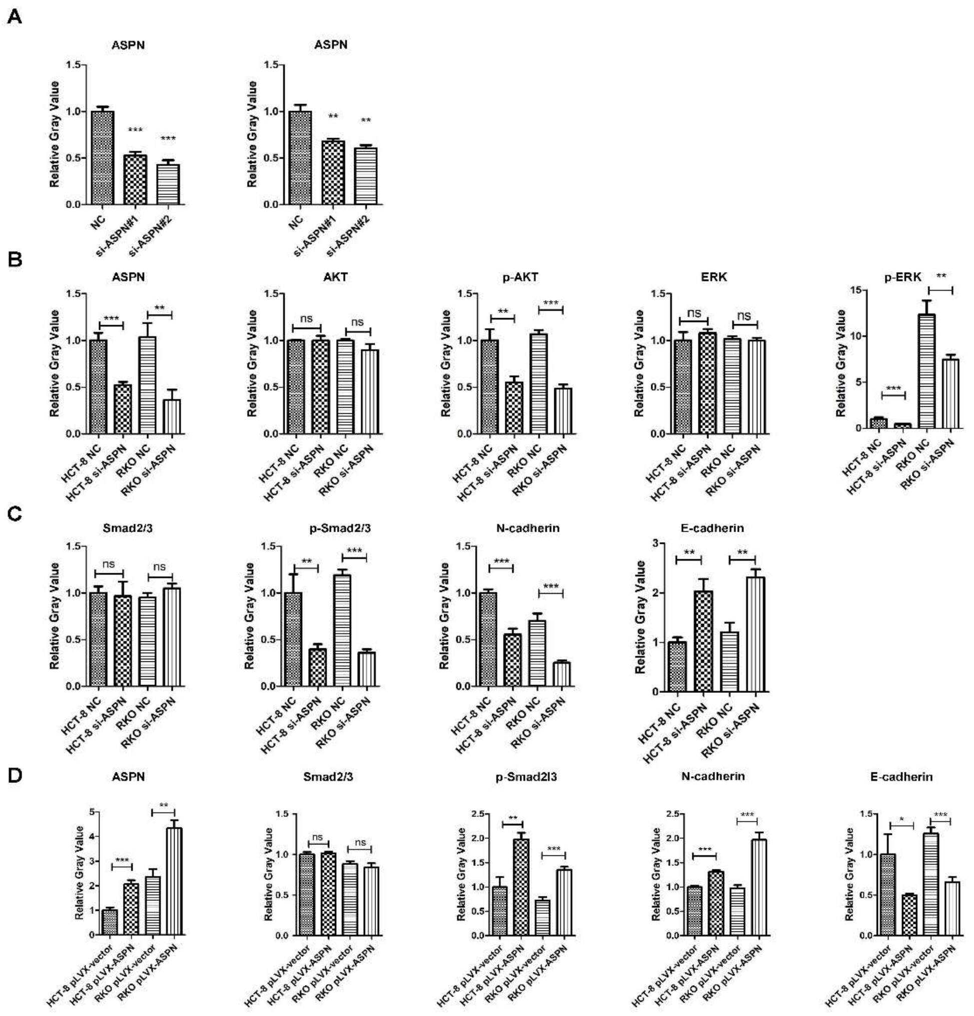

Supplementary Figure 2. Quantification of multiple western blot experiments.

A. Quantification of western blot experiments corresponding to Figure 2A.

B. Quantification of western blot experiments corresponding to Figure 3A.

C. Quantification of western blot experiments corresponding to Figure 3B.

D. Quantification of western blot experiments corresponding to Figure 3C.

Results are representative of three independent experiments. Values are the mean ± SD of the results. * p <0.05; **p <0.01; *** p <0.001.

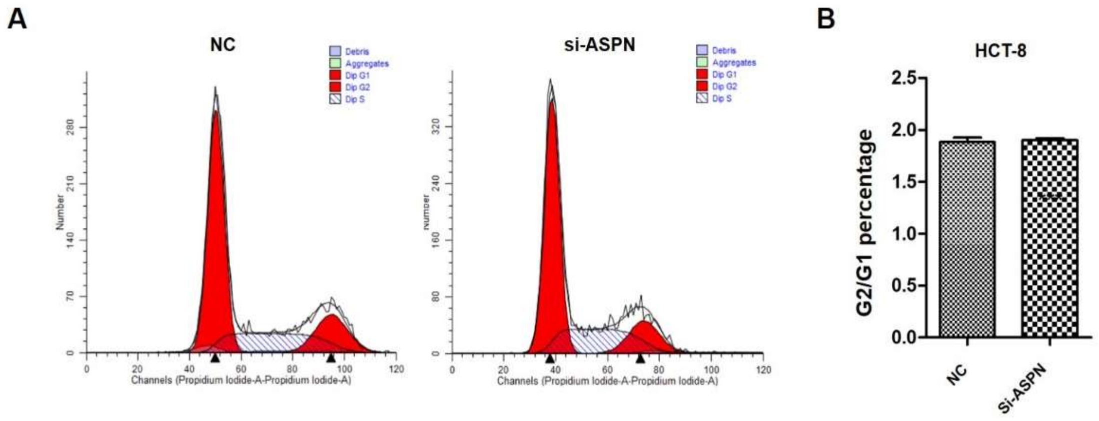

Supplementary Figure 3. ASPN did not affect cell cycle in CRC cells.

A. Cell cycle assays were detected by FACS applying HCT-8 cells after ASPN knockdown. B. Statistical analysis indicates no difference between si-ASPN treated HCT-8 cells and control. Results are representative of three independent experiments. Values are the mean ± SD of the results. * $p < 0.05$; ** $p < 0.01$; *** $p < 0.001$.

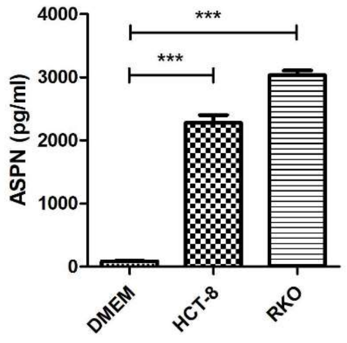

Supplementary Figure 4. Results of Elisa assays for secreted ASPN detection. Results indicates that both HCT-8 and RKO cell lines secrete ASPN protein. Results are representative of three independent experiments. Values are the mean ± SD of the results. * $p < 0.05$; ** $p < 0.01$; *** $p < 0.001$.

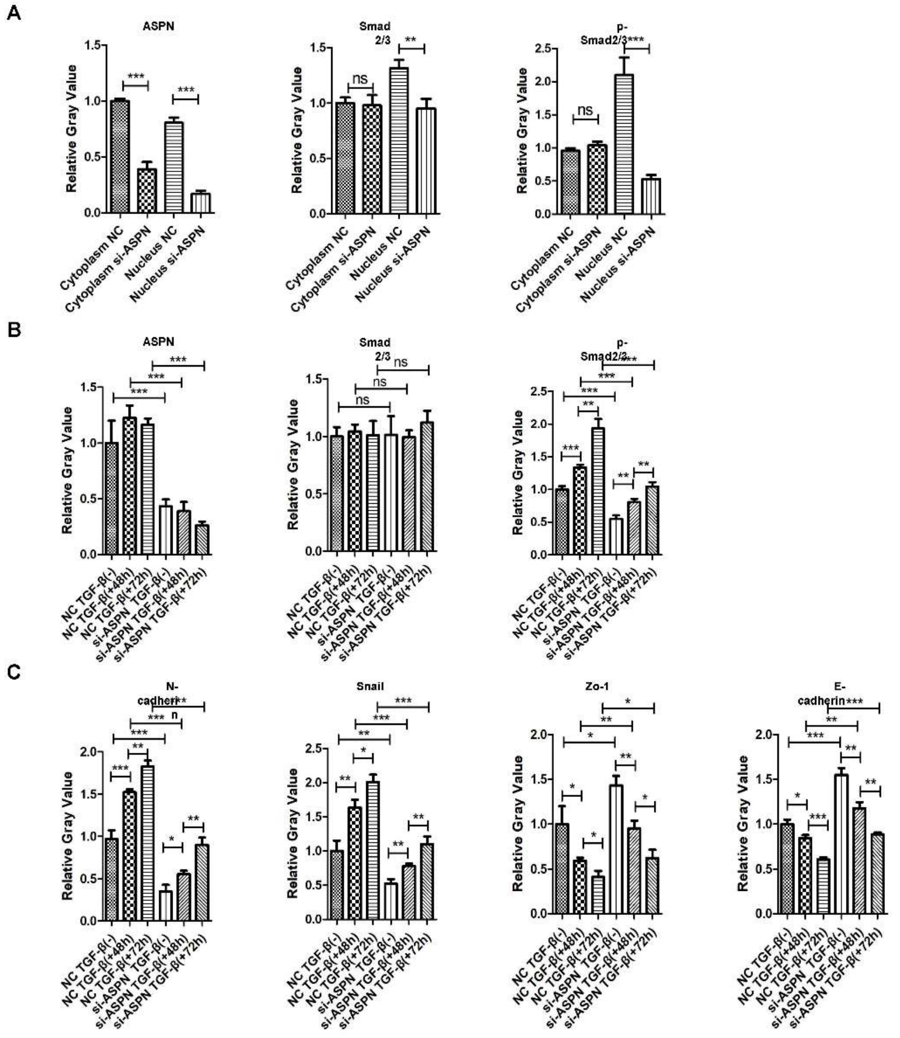

Supplementary Figure 5. Quantification of multiple western blot experiments.

A. Quantification of western blot experiments corresponding to Figure 5A.

B.C. Quantification of western blot experiments corresponding to Figure 6E.

Results are representative of three independent experiments. Values are the mean ± SD of the results. * $p < 0.05$; ** $p < 0.01$; *** $p < 0.001$.

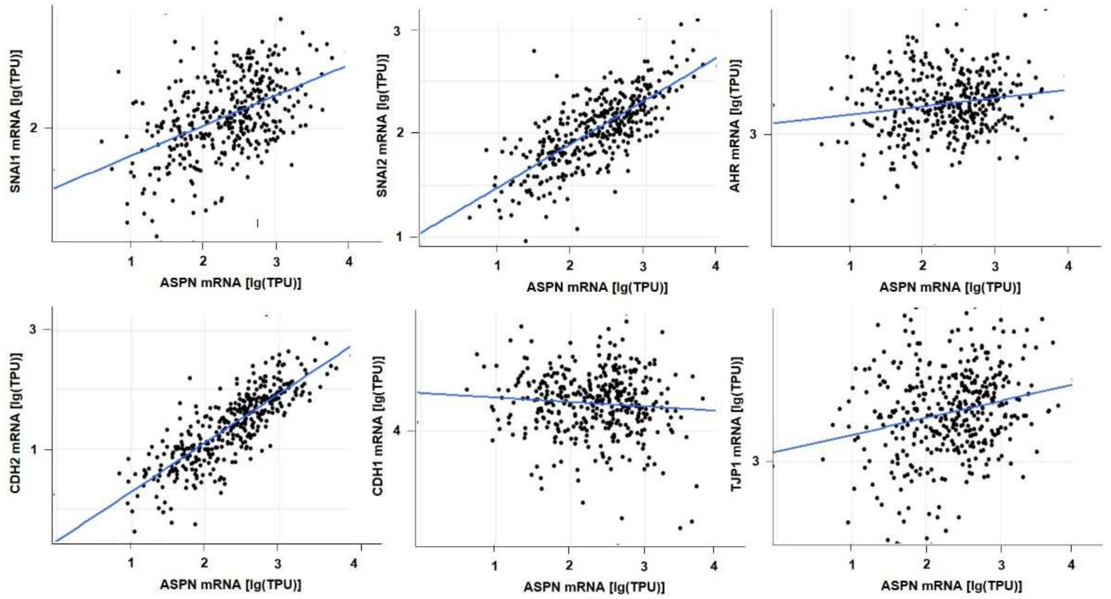

Supplementary Figure 6. In silico analysis indicated that ASPN was correlated with the expression of EMT markers in mRNA level.

ASPN positively correlates with the expression of SNAI1, SNAI2, AHR, CDH2 and TJP1, negatively correlates with that of CDH1 in mRNA level.

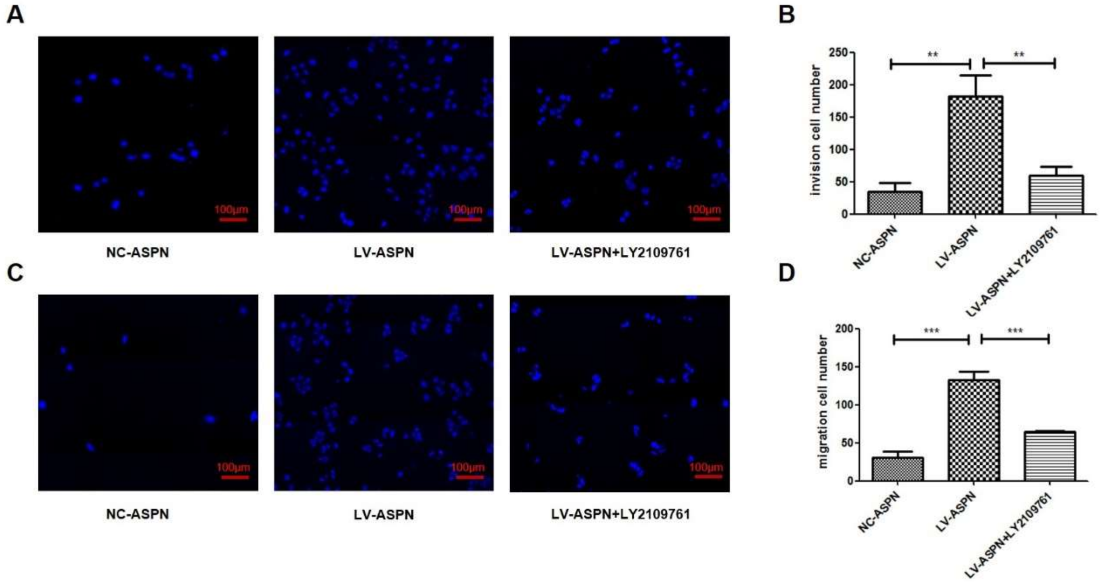

Supplementary Figure 7. The migration and invasion promoting function of ASPN could be reversed by TGF-βR inhibitor.

Transwell Assays were carried out to determine the function of TGF-βR antagonist LY2109761 in ASPN induced cell migration and invasion enhancement. A. ASPN overexpressed HCT-8 cells displayed an enhanced invasion ability. After treated with LY2109761 for 24h, the invasion promoting effect of ASPN were reversed. B. Statistical analysis of 3 independent invasion transwell experiments. C. ASPN overexpressed HCT-8 cells displayed an enhanced migration ability. After treated with LY2109761 for 24h, the migration promoting effect of ASPN were eliminated. D. Statistical analysis of 3 independent migration transwell experiments. Results are representative of three independent experiments. Values are the mean ± SD of the results. $^{**}p < 0.01$; $^{***}p < 0.001$.
