# Supplementary material for: Cytoplasmic Asporin promotes cell migration by regulating TGF-β/Smad2/3 pathway and indicates a poor prognosis in colorectal cancer
Source: Cell Death Dis. 2019 Feb 6;10(2):109. doi: 10.1038/s41419-019-1376-9 (PMC6365561; doi:10.1038/s41419-019-1376-9)
Supplement: Supplementary file 2 — Supplementary Tables [file 41419_2019_1376_MOESM2_ESM.pdf]

Supplementary Table 1. Information of primary antibodies in WB，IP and IF

Primary Antibody	Company	Cat.	Dilution Factor
GAPDH	Abbkine	A01020	WB 1:1000
ASPN	abcam	Ab58741	WB 1:1000
			IP 1:50
ASPN	SIGMA	HPA024230	IHC 1:200
			IF 1:50
AKT	invitrogen	44-609G	WB 1:1000
p-AKT	proteintech	66444-1-Ig	WB 1:1000
ERK	CST	4695T	WB 1:1000
p-ERK	CST	4370S	WB 1:1000
Smad2/3	Huabio	RT1566	WB 1:1000
			IP 1:50
p-Smad2/3	CST	8828S	WB 1:500
N-Cadherin	CST	13116	WB 1:1000
E-Cadherin	CST	3195	WB 1:1000
FLAG	SIGMA	F1804	IP 1:50
Lamin B1	CST	9087S	WB 1:1000
Snail	CST	3879	WB 1:1000
Zo-1	CST	8193T	WB 1:1000
GFP	abcam	Ab290	WB 1:1000

Supplementary Table 2. Information of primers used in RT-qPCR.

Primer	Sequence	Base number
MMP2 F	TACAGGATCATTGGCTACACACC	23
MMP2 R	GGTCACATCGCTCCAGACT	19
MMP9 F	TGTACCGCTATGGTTACACTCG	22
MMP9 R	GGCAGGGACAGTTGCTTCT	19
TCF4 F	GGCTATGCAGGAATGTTGGG	20
TCF4 R	GTTCATGTGGATGCAGGCTAC	21
TNC F	TCCCAGTGTTCGGTGGATCT	20
TNC R	TTGATGCGATGTGTGAAGACA	21
AHR F	ACATCACCTACGCCAGTCG	19
AHR R	CGCTTGGAAGGATTTGACTTGA	22
GLI2 F	CTGCCTCCGAGAAGCAAGAAG	21
GLI2 R	GCATGGAATGGTGGCAAGAG	20
ZEB1 F	GATGATGAATGCGAGTCAGATGC	23
ZEB1 R	ACAGCAGTGTCTTGTTGTTGT	21
GAPDH F	GGAGCGAGATCCCTCCAAAAT	21
GAPDH R	GGCTGTTGTCATACTTCTCATGG	23

Supplementary Table 3. Associations between ASPN expression and clinicopathological factors in 88 CRC patients.

Variables	ASPN		χ^2	*P*-Value
	Positive (63)	Negative (25)		
**Gender**			0.1945	0.6592
Male	32	14		
Female	31	11		
**Age (years)**			2.7378	0.0980
≥ 65	35	9		
< 65	28	16		
**Tumor Size (cm)**			1.2590	0.2618
≥5	32	16		
< 5	31	9		
**T stage**			1.2141	0.2705
T1, T2	20	5		
T3, T4	43	20		
**Lymph Node Metastasis**			1.0980	0.2947
Negative	30	15		
Positive	33	10		
**Distant Metastases**			1.2724	0.2593
Negative	50	17		
Positive	13	8		
**Pathological Stage**			0.0826	0.7739
I-II, II	46	19		
II-III, III	17	6		
**Clinical Stage**			0.0104	0.9187
1, 2	32	13		
3, 4	31	12		
